# Sequence Variation in Rhoptry Neck Protein 10 Gene among *Toxoplasma gondii* Isolates from Different Hosts and Geographical Locations

**Published:** 2017

**Authors:** Yu ZHAO, Donghui ZHOU, Jia CHEN, Xiaolin SUN

**Affiliations:** 1. College of Veterinary Medicine, Gansu Agricultural University, Lanzhou, China; 2. State Key Laboratory of Veterinary Etiological Biology, Key Laboratory of Veterinary Parasitology of Gansu Province, Lanzhou Veterinary Research Institute, Chinese Academy of Agricultural Sciences, Lanzhou, China

**Keywords:** *Toxoplasma gondii*, Toxoplasmosis, Rhoptry neck protein 10 (TgRON10), Sequence variation

## Abstract

**Background:**

*Toxoplasma gondii,* as a eukaryotic parasite of the phylum Apicomplexa, can infect almost all the warm-blooded animals and humans, causing toxoplasmosis. Rhoptry neck proteins (RONs) play a key role in the invasion process of *T. gondii* and are potential vaccine candidate molecules against toxoplasmosis.

**Methods:**

The present study examined sequence variation in the rhoptry neck protein 10 (TgRON10) gene among 10 *T. gondii* isolates from different hosts and geographical locations from Lanzhou province during 2014, and compared with the corresponding sequences of strains ME49 and VEG obtained from the ToxoDB database, using polymerase chain reaction (PCR) amplification, sequence analysis, and phylogenetic reconstruction by Bayesian inference (BI) and maximum parsimony (MP).

**Results:**

Analysis of all the 12 TgRON10 genomic and cDNA sequences revealed 7 exons and 6 introns in the TgRON10 gDNA. The complete genomic sequence of the TgRON10 gene ranged from 4759 bp to 4763 bp, and sequence variation was 0–0.6% among the 12 *T. gondii* isolates, indicating a low sequence variation in TgRON10 gene. Phylogenetic analysis of TgRON10 sequences showed that the cluster of the 12 *T. gondii* isolates was not completely consistent with their respective genotypes.

**Conclusion:**

TgRON10 gene is not a suitable genetic marker for the differentiation of *T. gondii* isolates from different hosts and geographical locations, but may represent a potential vaccine candidate against toxoplasmosis, worth further studies.

## Introduction

Toxoplasmosis caused by *Toxoplasma gondii* is one of the most common parasitic zoonoses worldwide, with a wide range of hosts including almost all warm-blooded animals (1–4). Global epidemiologic studies of toxoplasmosis indicate that overall 33% people infected the *T. gondii* (1, 5).

The rhoptry is a subcellular organelle of apicomplexan parasites. Rhoptry neck proteins (RONs) are secreted by rhoptry for the formation of moving junction (MJ), which plays an important role in the invasion of *T. gondii* (6). Therefore, the research on RONs can help us to better understand the pathogenic mechanism of *T. gondii* and explore the effective approaches for prevention and treatment of toxoplasmosis. Some studies indicate that RONs are concerned with *T. gondii* invasion, so they are underlying candidate antigens of DNA vaccine to against *T. gondii* (7). Rhoptry neck protein 10 (TgRON10) is a component of the newly identified RON9/RON10 complex in *T. gondii*, related with development of *T. gondii* in intestinal epithelial cells (8).

However, little is known about sequence variation in TgRON10 gene among *T. gondii* isolates of different genotypes. The aim of this study was to examine the sequence variation in TgRON10 genes among *T. gondii* isolates from different hosts and geographical locations, and to assess whether the TgRON10 gene sequence may represent a new marker for studying *T. gondii* population genetic structures.

## Materials and Methods

### *T. gondii* isolates

Ten *T. gondii* isolates originating from different hosts and geographical locations were used in this study from Lanzhou Province during 2014 (Table 1), and genomic DNA (gDNA) of these *T. gondii* isolates was prepared and genotyped in our previous studies (9–12). Two corresponding sequences of strains ME49 (ToxoDB: TGME49_261750) and VEG (ToxoDB: TGVEG_261750) were obtained from the ToxoDB database.

**Table 1:** Details of *Toxoplasma gondii* isolates used in the present study

**Strain**	**Host**	**Geographical origin**	Genotype*
**GT1**	Goat	United States	Reference, Type I, ToxoDB #10
**RH**	Human	France	Reference, Type I, ToxoDB #10
**CTG**	Cat	United States	Reference, Type III, ToxoDB #2
**VEG**	Human	United States	Reference, Type III, ToxoDB #2
**MAS**	Human	France	Reference, ToxoDB #17
**TgCatBr5**	Cat	Brazil	Reference, ToxoDB #19
**TgCatBr64**	Cat	Brazil	Reference, ToxoDB #111
**SH**	Human	Shanghai, China	Type I, ToxoDB #10
**ME49**	Sheep	United States	Type II, ToxoDB #1
**Prugniaud (PRU)**	Human	France	Type II, ToxoDB #1
**PYS**	Pig	Panyu, China	ToxoDB #9
**TgWtdSc40**	Deer	USA	Type 12, ToxoDB #5

* based on previous genotyping results (9–12)

### PCR amplification

gDNA of individual isolates was used as template for the amplification of the entire TgRON10 gene sequences. A pair of oligonucleotide primers: TgRON10F (forward primer, 5′-ATgCCTgAGGTTAACTgC-3′) and TgRON10R (reverse primer, 5′-TTAAGAAGAGTCTTCTgTCGC-3′) were designed based on the TgRON10 gene sequence of *T. gondii* ME49 strain available in ToxoDB database (TgME49_261750). PCR reactions were carried out in 25 μL containing 10 mM Tris-HCl (pH 8.4), 50 mM KCl, 3 mM MgCl_2_, 250 μM each of dNTP, 0.2 μM of each primer, 100–200 ng of template DNA, and 0.25 U La *Taq* polymerase (TaKaRa). The PCR reaction was carried out in a thermocycler (Bio-Rad) with an initial denaturation at 94 °C for 4 min, followed by 37 cycles of 94 °C for 30 sec (denaturation), 67.5 °C for 30 sec (annealing), 72 °C for 5 min (extension), and a final extension of 72 °C for 10 min. A negative control sample without gDNA was included in each PCR reaction. Each amplicon (6 μl) was examined on 1% (w/v) agarose gel to assess amplification efficiency. Sizes of TgRON10 PCR products were estimated by using a DNA marker (DL2000 plus, TAKARA), and photographed using a gel documentation system (UVP GelDoc-ItTM Imaging System, Cambridge, U.K.).

### Sequencing of the TgRON10 amplicons

Positive TgRON10 amplicons were purified using the spin columns according to the manufacturer’s recommendations (Wizard™ PCR-Preps DNA Purification System, Promega, USA), ligated into pGEM-T-Easy vector (Promega), and then transformed into the JM109 competent cells (Promega, USA). Following the screening by PCR amplification, the positive colonies were sequenced by Shanghai Songon Biological Engineering Biotechnology Company.

### Sequence analysis and phylogenetic reconstruction

The obtained TgRON10 gene sequences from different *T. gondii* strains were aligned using the computer program ClustalX 1.83 (13), and sequence variation was determined among the examined *T. gondii* strains. Phylogenetic reconstructions based on the complete sequences of TgRON10 gene among different *T. gondii* strains was performed by Bayesian inference (BI) and maximum parsimony (MP) using *Neospora caninum* (GenBank accession No. FR823389.1) as the out-group. BI analyses were conducted with four independent Markov chains run for 10000000 metropolis-coupled MCMC generations, sampling a tree every 10000 generations in MrBayes 3.1.1 (14). The first 250 trees were omitted as burn-ins and the remaining trees were used to calculate Bayesian posterior probabilities (PP). MP analysis was performed using PAUP* 4.0b4a (15), with indels treated as missing character states. Overall, 1000 random addition searches using TBR were performed for each MP analysis. Bootstrap probability (BP) was calculated from 1000 bootstrap replicates with 10 random additions per replicate in PAUP. Phylograms were drawn using the Tree View program ver. 1.66 (16).

## Results

**Fig. 1:**
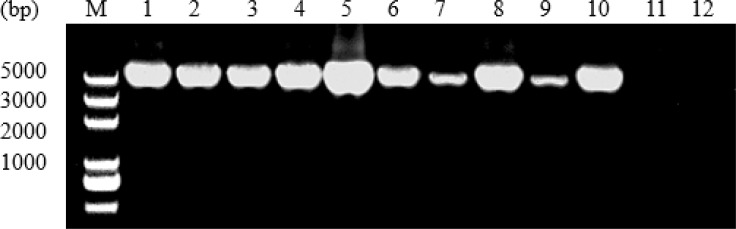
Electrophoresis of PCR amplification of RON10 from different *T. gondii* strains M. DL5000 DNA Marker 1. RH 2. GT1 3. PYS 4. CTG 5. PRU 6. MAS 7. TgCatBr5 8. TgCatBr64 9. SH 10. TgWtdSc40 11. Host control 12. Negative control

**Fig. 2:**
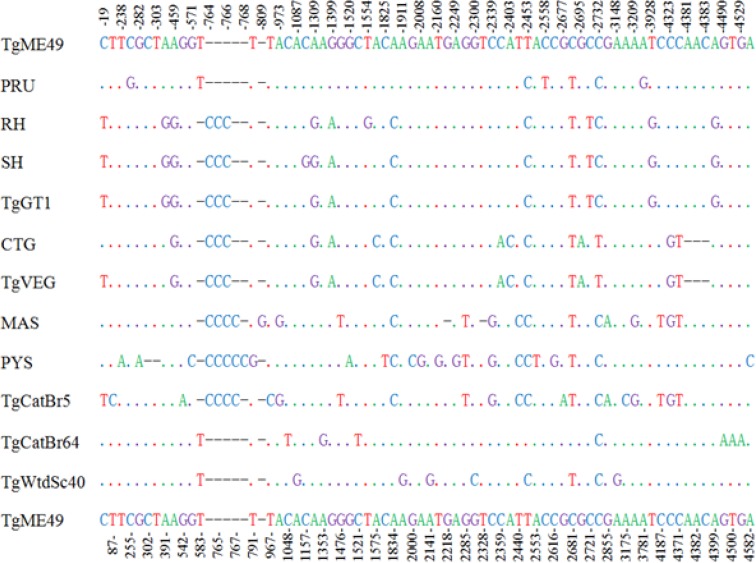
Multiple alignment analyses for nucleotides sequences of *Toxoplasma gondii* RON10 gene Point (.) stands for identical nucleotide, dash (-) indicates nucleotide deletions in comparison to that of *T. gondii* ME49 strain (upper and bottom lines), and the numbers indicate the variable sequence positions for nucleotide

PCR amplification of TgRON10 gene from different *T. gondii* isolates produced a single band of approximately 4600 bp in length on agarose gel (Fig. 1). Positive TgRON10 amplicons were purified and ligated with clone vector, and then transformed into the competent cells. Following the screening by PCR amplification, the positive colonies were sequenced from both directions. The obtained entire genomic sequences of TgRON10 gene was 4759 bp in length for the strains CTG and VEG, 4762 bp for the strains GT1, MAS, RH, SH and PYS, 4763 bp for the strain TgCatBr5, and 4760 bp for the other four strains. Analysis of all the 12 TgRON10 complete genomic sequences revealed 7 exons and 6 introns in the TgRON10 gene, the A+T contents varied from 48.43% to 48.61% in the entire sequence. There were 124 nucleotide position variations in the entire genomic sequences (Fig. 2). A total of 55 nucleotide position variations in exons with a distribution of two deletions of 3 bp in the sequence of strains CTG and VEG, 40 transitions (C<–>T, A<–>C, and A<–>G) and 9 transversions (A<–>T and C<–>G) (R=transition/transversion=4.4) (Table 2). In addition, there were 124 nucleotide position variations in the intron among the 12 examined *T. gondii* isolates, including 42 deletions, 75 transitions (C<–>T, T<–>G, A<–>C, and A<–>G) and 7 transversions (A<–>T and C<–>G) (Table 3).

**Table 2:** Characteristics of *Toxoplasma gondii* TgRON10 gene sequences including exons

**Item**	**TgRON10 gDNA**	**TgRON10 cDNA**	**TgRON10 exons**						
			**First**	**Second**	**Third**	**Forth**	**Fifth**	**Sixth**	**Seventh**
Length (bp)	4759–4763	2505–2508	259	455	250	80	476	218	767–770
T+A (%)	48.43–48.61	45.14–45.37	46.72–47.10	44.62–45.27	46.40–46.80	45.00	44.12–44.33	46.33–46.79	44.42–44.81
Transition	115	40	6	5	10	0	6	1	12
Transversion	16	9	2	1	0	1	0	0	5
R	7.4	4.4	3	5	/	/	/	/	2.4
Distance (%)	0–0.6	0–0.6	0–1.2	0–0.7	0–1.2	0–1.3	0–0.8	0–0.5	0–0.8

R=transition/transversion.

**Table 3:** Characteristics of *Toxoplasma gondii* TgRON10 gene intron sequences

***Item***	***TgRON10 introns***					
	**First**	**Second**	**Third**	**Forth**	**Fifth**	**Sixth**
Length (bp)	551–555	434	522–524	206	324	213
T+A (%)	51.36–52.09	51.84–52.30	53.24–54.01	49.51–50.97	50.00	53.99–54.46
Transition	11	18	24	19	0	3
Transversion	1	0	5	1	0	0
R	11	/	4.8	19	/	/
Distance (%)	0–0.7	0–1.2	0–1.7	0–1.5	0	0–0.5

R=transition/transversion

## Discussion

In the present study, the alignment of TgRON10 entire genomic sequences showed that sequence variation were 0–0.6% in all examined strains. The deduced amino acid sequence analysis showed the presence of 30 substitutions and two deletions among the 12 examined *T. gondii* isolates, which is lower than that in ROP7 and ROP13 genes (17, 18). Variation in TgRON10 sequences among the examined *T. gondii* isolates was slightly low, and similar results were found in previous studies, such as PLP1 (19), MIC13 (20) and other genes among the clonal lineages of *T. gondii* (21). In summary, our data indicated the existence of low sequence variation in TgRON10 gene among different *T. gondii* isolates, thus it is not a suitable genetic marker for genotyping studies in *T. gondii*. However, due to the high identity in different *T. gondii* isolates, RON10 gene may be an ideal immune effector molecule against different *T. gondii* isolates infection, worth further study.

**Fig. 3:**
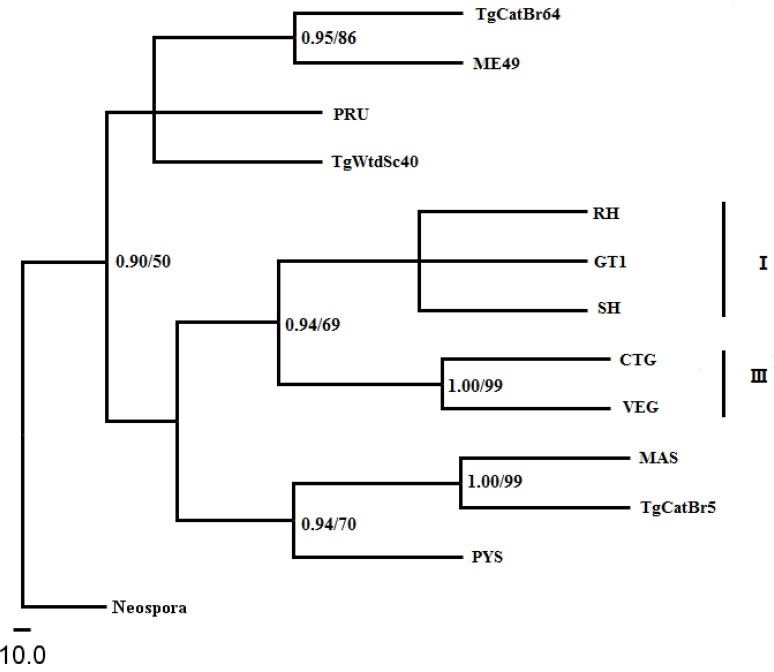
Phylogenetic relationships of *Toxoplasma gondii* isolates from different hosts and geographical locations inferred by Bayesian inference (BI) and maximum parsimony (MP) analyses based on the TgRON10 gene sequences using *Neospora caninum* (GenBank accession No. FR823389.1) as outgroup. The numbers along branches indicate bootstrap values resulting from different analysis in the order: BI/MP. I and III represented two major clonal lineages (Type I and III) of *T. gondii* isolates.

Phylogenetic analysis using BI and MP based on TgRON10 sequence of all 12 *T. gondii* strains has shown that the two major clonal lineages (Type I and III) can be differentiated (Fig. 3). All the Type I strains SH, GT1, and RH clustered together. Two Type III strains CTG and VEG grouped together. However, the two Type II strains PRU and ME49 were separated strains representing other genotypes (Fig. 3).

## Conclusion

This study revealed the existence of low sequence variability in TgRON10 gene among the examined *T. gondii* isolates from different hosts and geographical locations. TgRON10 gene may not be a suitable marker for population genetic studies of *T. gondii* isolates but may represent a potential vaccine candidate against *T. gondii* infection.

## References

[1] Tenter AM Heckeroth AR Weiss LM *Toxoplasma gondii*: from animals to humans. Int J Parasitol. 2000; 30(12–13):1217–58. 11113252 10.1016/s0020-7519(00)00124-7 PMC3109627

[2] Montoya JG Liesenfeld O Toxoplasmosis. Lancet. 2004; 363(9425):1965–76. 15194258 10.1016/S0140-6736(04)16412-X

[3] Dubey JP Toxoplasmosis of animals and humans. Parasit Vectors. 2010; 3(1):112.

[4] Zhou P Chen Z Li HL *Toxoplasma gondii* infection in humans in China. Parasit Vectors. 2011; 4:165. 21864327 10.1186/1756-3305-4-165 PMC3174123

[5] Hill D Dubey JP *Toxoplasma gondii*: transmission, diagnosis and prevention. Clin Microbiol Infect. 2002; 8(10):634–40. 12390281 10.1046/j.1469-0691.2002.00485.x

[6] Bradley PJ Ward C Cheng SJ Proteomic analysis of rhoptry organelles reveals many novel constituents for host-parasite interactions in *Toxoplasma gondii*. J Biol Chem. 2005; 280(40):34245–58. 16002398 10.1074/jbc.M504158200

[7] Rashid I Hedhli D Moiré N Immunological responses induced by a DNA vaccine expressing RON4 and by immunogenic recombinant protein RON4 failed to protect mice against chronic toxoplasmosis. Vaccine. 2011; 29(48):8838–46. 21983362 10.1016/j.vaccine.2011.09.099

[8] Lamarque MH Papoin J Finizio AL Identification of a new rhoptry neck complex RON9/RON10 in the Apicomplexa parasite *Toxoplasma gondii*. PloS One. 2012; 7(3): e32457. 22427839 10.1371/journal.pone.0032457 PMC3299665

[9] Zhou P Zhang H Lin RQ Genetic characterization of *Toxoplasma gondii* isolates from China. Parasitol Int. 2009; 58(2): 193–5. 19567233 10.1016/j.parint.2009.01.006

[10] Weiss LM Dubey JP Toxoplasmosis: A history of clinical observations. Int J Parasitol. 2009; 39(8): 895–901. 19217908 10.1016/j.ijpara.2009.02.004 PMC2704023

[11] Su C Shwab EK Zhou P Zhu XQ Dubey JP Moving towards an integrated approach to molecular detection and identification of *Toxoplasma gondii*. Parasitology. 2010; 137(1):1–11. 19765337 10.1017/S0031182009991065

[12] Huang SY Cong W Zhou P First report of genotyping of *Toxoplasma gondii* isolates from wild birds in China. J Parasitol. 2012; 98(3):681–2. 22263675 10.1645/GE-3038.1

[13] Thompson JD Gibson TJ Plewniak F The CLUSTAL_X windows interface: flexible strategies for multiple sequence alignment aided by quality analysis tools. Nucleic Acids Res. 1997; 25(24):4876–82. 9396791 10.1093/nar/25.24.4876 PMC147148

[14] Ronquist F Huelsenbeck JP MrBayes 3: Bayesian phylogenetic inference under mixed models. Bioinformatics. 2003; 19(12):1572–4. 12912839 10.1093/bioinformatics/btg180

[15] Swofford DL PAUP: Phylogenetic Analysis Using Parsimony (and Other Methods). Sunderland MA: Sinauer Associates; 2002.

[16] Page RD TreeView: an application to display phylogenetic trees on personal computers. Comput Appl Biosci. 1996; 12(4):357–8. 8902363 10.1093/bioinformatics/12.4.357

[17] Wang PY Lu P Xu MJ Genetic diversity among *Toxoplasma gondii* isolates from different hosts and geographical locations revealed by analysis of ROP13 gene sequences. Afr J Biotechnol. 2012; 11(25): 6662–5.

[18] Zhou Y Lu P Xu MJ Sequence variation in TgROP7 gene among *Toxoplasma gondii* isolates from different hosts and geographical regions. Afr J Biotechnol. 2012; 11(25): 6658–61.

[19] Yan HK Song HQ Zhou Y Sequence variation in perforin-Like protein 1 gene among six *Toxoplasma gondii* strains. J Anim Vet Adv. 2011; 10(17): 2244–47.

[20] Ren D Zhou DH Xu MJ Zhou Y Sequence variation in *Toxoplasma gondii* MIC13 gene among isolates from different hosts and geographical locations. Afr J Microbiol Res. 2012; 6(6): 1333–37.

[21] Khan A Taylor S Ajioka JW Rosenthal BM Sibley LD Selection at a single locus leads to widespread expansion of *Toxoplasma gondii* lineages that are virulent in mice. PLoS Genet. 2009; 5(3):e1000404. 19266027 10.1371/journal.pgen.1000404 PMC2644818

